# Some Enquiries in the Province of Kemaon, Relative to Geology; Including an Enquiry into the Causes of Goitre

**Published:** 1839-07

**Authors:** 


Art. V.

1. *Some Enquiries in the Province of Kemaon, relative to Geology; including an enquiry into the causes of Goitre.* By John M'Clelland, Assistant Surgeon, Bengal Army.—*Calcutta*, 1835. 8vo, pp. 384.

2. *Some account of the Bronchocele of Nipaul, and of the Cis and Trans-Himalayan Regions.* By the late M. J. Bramley, Principal of the Calcutta Medical College, (Transactions of the Medical and Physical Society of Calcutta, Vol. vi.)—*Calcutta*, 1833.

3. *Treatise on English Bronchocele, with a few Remarks on the use of Iodine and its Compounds.* By James Inglis, m.d.—*London*, 1838. 8vo, pp. 95.

Mr. M'Clelland has prefixed to the enquiry into the nature and causes of Bronchocele, which constitutes the third part of his work on the geology, natural history, and medical statistics of the sub-Himalayan district of Kemaon, a maxim of Lord Bacon, the truth of which we hesitate to admit, as its adoption would go far to remove medicine from its place amongst the sciences. If "true knowledge is the knowledge of causes," how little do we know of the nature of any disease, or of those remedies on which we place our firmest confidence? But if Laennec, who confesses that the causes of disease are almost entirely unknown, was yet able to throw such clear light on many of the most important disorders to which the human frame is subject, we may pause before we accept the dictum of the founder of inductive philosophy in its application to medicine; and zealously pursue the course of minute and accurate observation, although it may not actually lead us to the knowledge of causes. When we have learned that iodine removes a goitre of a particular kind, and fails to do so when prescribed in a different form of the disease, and when we have laid down rules by which these varieties are distinguished, we have acquired a species of knowledge as true, if not so extensively applicable, as if we could explain the vital or chemical actions by which this singular substance exerts its influence over these mysterious tumours.

While we think it right to make these observations, on the error of applying some of the principles that ought to guide physical researches to medical enquiries, we cannot appreciate too highly the labours of those who, like Mr. M'Clelland, have set themselves the irksome task of carefully collecting facts by which the cause of any diseased action may in time be ascertained. Bronchocele is a disease which has always appeared to us to be particularly interesting, and the study of which ought to be valuable, not only because its external properties and site give it a distinct and positive character which very few other diseases possess, but also as being evidently connected with certain physical circumstances in the countries in which it prevails, which admit of being determined, and through the study of which we arrive at facts not only sufficient to explain the causes of this disease, but also to throw light on many important questions relative to the influence of physical agents on animal life.

Nor will limited enquiries, confined to those countries with which our local circumstances may bring us acquainted, be sufficient to enable us to arrive at any conclusions that will bear the test of time and enlarged research; on the contrary, we must trace its prevalence, not only through our own inland districts, along the valleys of the Alps and the Pyrenees, but pursue the enquiry on the elevated plains of Columbia, amongst the lofty and damp valleys of Sumatra; and, as displayed in the works before us, in the deep and narrow dells of Kemaon, the great almost tropical valley of Nipaul, and the lofty table lands of Tibet and Tartary. Before the philosophy of medicine can deserve the name, pathology must be investigated in all the various climates of the world; and the profession must cease to turn a deaf ear to the researches of those who have to contend with disease, under circumstances of climate and modes of life very different from those in which they are met with in England or in Europe. In treating of bronchocele we may be allowed to use a geological illustration, and compare such a restricted course of study to that of the followers of Werner, who arranged rocks and speculated on their formation, from the appearances presented to his examination in a corner of Germany. Of the necessity for this enlarged enquiry, no disease affords a better example than goitre, which, from the time of Pliny till Mr. Marsden published his History of Sumatra, was ascribed to the use of snow-water, a theory at once overthrown by his account of the complaint in the moist valleys of that great island, in which snow never falls.* We would, therefore, urge those of our readers, who reside in foreign countries having peculiar climates, geological structure, or other physical distinctions, to spare no labour in accumulating facts, and in submitting them to the profession, assured that, sooner or later, they will meet with attention, however much they may be overlooked for a time by the periodical press, which too often sacrifices matters of great and permanent importance to the pressure of subjects of mere temporary interest. We know that vast accumulations, the result of most careful observation, have been lost to the public, by a feeling on the part of our countrymen practising abroad, that their labours would either be overlooked or contemned by the retailers of medical information; and we fear that too much cause has been given for the impression prevalent among them, that except they lend their views to support some fashionable system of practice, however injurious, or flatter some reigning prejudice, they will either be passed over unnoticed or unjustly condemned. Had the methods of practice followed in India early in the present century, and the success attending them been given to the public, we are satisfied that much severe suffering and loss of life, caused by the successful advocacy of a rude and indiscriminating use of the lancet, large doses of calomel, and the abuse of drastic purgatives would have been prevented.

Mr. M'Clelland's work is divided into three parts: the first is dedicated to a detailed physical and geological description of the district of Kemaon; the second to its zoology and meteorology; and the third, consisting of 130 pages, to the investigation of the causes of bronchocele, and contains the most minute statistical details on this subject with which we are acquainted. The geological portion of the work, although much tinctured with the doctrines of the Wernerian school (which Mr. M'Clelland has since renounced), and with an error natural to a pupil of that school, of

* See also Phil. Trans., 1784.

attempting, on very insufficient grounds, to reduce to European types the rock-formations of the Himalayas, yet abounds with those minute details of structure and composition, so much neglected by many of the best English geologists, and so essential to the physician, who would examine into any alleged connexion between the nature of the rocks and the diseases prevalent amongst the inhabitants who live on them. In this view, Mr. M'Clelland's geological researches may be considered as introductory to his enquiry into the cause of goitre; and we cannot but admire the ardent zeal which enabled him, while actively engaged in professional duties, to investigate the structure of nearly 1000 square miles of a wild mountainous country, intersected by deep unhealthy valleys. The overcoming such difficulties deserves our warmest praise; and it reflects credit on the present Indian government, to have rewarded his unaided exertions, by appointing him a member of the committee to explore the tea-country to the N.E. of Bengal, and secretary to the recently constituted commission for investigating the mineral resources of our eastern empire. It is, however, to be regretted, that Mr. M'Clelland did not publish his researches on goitre in a separate form, as few medical readers will be able to procure, or have time to peruse, the copious details of a non-medical nature that constitute the bulk of the volume. We shall therefore give a copious abstract of the important facts contained in the work, in the hope that it may induce those of our readers, either in this country or on the continent, who are favorably situated, to follow Mr. M'Clelland's example, and to communicate accurate statistical details, accompanied with geological and meteorological observations, and information on the habits of life of the inhabitants of affected districts, with the view of testing the truth of his conclusions.

The snowy peaks of the Himalayas, from 18 to 25,000 feet high, bound the district of Kemaon to the north, while the rich plains of Rohilkund extend from the opposite side of the province to the Ganges, the intervening country being occupied by precipitous ranges of mountains from 4 to 8,000 feet in height, inclosing deep and narrow valleys, and giving passage, by narrow and tremendous defiles, to the tributaries of the Gogra, one of the great feeders of the Ganges. These streams are sometimes seen but oftener heard rushing in furious torrents down the river-valleys which divide the mountain groups from each other, and which are usually from 2 to 5,000 feet below the adjoining summits. Mr. M'Clelland divides the province into three districts; the first extending from the Burmodea pass, by which the road enters the mountains from the low and moist tract, of which Heber has given so appalling a picture, to Balket, and is composed of a rugged group of mountains, 8,000 feet in height, mostly composed of clayslate, and covered with dense forest.* This tract is nearly uninhabited, and the deep valleys near the plains cannot be entered during six months of the year, on account of the deadly fevers that then prevail.

Mr. M'Clelland was not able to prosecute his researches to any extent amongst the few migratory inhabitants of this dismal tract, who spend

* It may be proper to state that, by some mistake, Mr. M'Clelland mentions extensive beds of gypsum, which turned out to be a rock of a very different kind. See paper, by the author, Journal of the Asiatic Society of Bengal, August, 1837.

the summer on the mountains, and when the sickly season is past, return to the valleys to avoid the rigors of winter; we shall, therefore, proceed to an analysis of the remainder of the paper. In the following extract he states his opinions as to the cause of goitre, and the course of enquiry by which he was led to adopt them.

"During the course of the enquiries into the geology of the province, contained in the first part of the work, I was struck with the frequency of goitre in one portion of the district, while the other was almost exempt from the complaint, although an equality of moral as well as physical circumstances appeared to affect the whole. The external Alpine characters of the province are the same in every part, the inhabitants all belong to the same tribes of Hindoos, and are subject to fewer irregularities in their mode of life than any other people in the world. In such a field there could be little merit in eliciting highly important facts connected with this intricate subject.

"That portion of Kemaon which lies on the south of the Ramesa river is composed of siliceous and argillaceous rocks of the primitive class. In the centre of this ridge there are numerous small valleys, some of them 7,000 and others as low as 3,000 feet above the sea, inhabited by persons who, some to avoid the winter's cold of their native mountains and some to avail themselves of pasture for their cattle, descend into the plains, and are absent from their villages five months of every year. From enquiries which I made amongst these people, I found them to be affected with goitre in the proportion of 1 in 500; but, as they do not constantly reside in the mountains, they are excluded from the more minute statistic details.

"The north-eastern acclivity of this chain is intersected by numerous deep river valleys and ravines, as well as by low mountain ridges, which afford a climate more congenial to the feelings and wants of the inhabitants, who here reside constantly in their villages. Of these villages 46 have been visited; but two of their number, having been occupied for only three or four years, are excluded from the general view; so that the number of villages on the south of the Ramesa river, which we are now to consider, amount to 43, and contain a population of 3,700; of this number I found only 17 persons affected with goitre, and these were exclusively adults. The different localities of these villages are as diverse as can well be imagined. Some are erected on narrow ridges, others in deep valleys, surrounded by abrupt and lofty mountains; others on rugged declivities, between lofty peaks on the one side and dark ravines on the other, into some of which the sun can scarcely penetrate. The different altitudes of these villages vary from 2,000 to 6000 feet.

"Let us now cross the Ramesa river, and enter the district of Shore, whose geological distinctions have been pointed out in a former part of this work; and we find that one eighth part of the people are affected with goitre. Yet the whole of the inhabitants of the province are equally circumstanced with respect to religion; they intermarry, have the same customs, and are affected alike by moral and political influences; and finally, the tract in which the disease prevails is the richest and most fertile portion of the province.

The natives themselves impute to the quality of the waters a powerful influence over their health; and when it is recollected that water and farinaceous vegetables constitute the chief diet of the Hindoos, any impurity of that fluid would produce effects more readily upon them than on persons whose food and habits are less simple; but whether they are right or wrong, in ascribing the prevalence of goitre to the impurity of particular waters, I shall not here stop to enquire. A subject on which so many conflicting opinions exist requires to be elucidated by such facts as, from their number, force, and simplicity, can lead to no erroneous interpretation; and in collecting these facts, the method I adopted, on observing the prevalence of the disorder in one great section of the district, and its absence in another, was to mark the physical characters by which these places were distinguished from each other. The consequence was, a perfect agreement in external aspect, altitude, and chimatology, but a very marked difference in their geognostic relations: and this distinction, which was even traced down to the very villages in which the disease is found, with such perfect nicety, as to enable one almost to predict *a priori*, on examining the rocks of a neighbourhood, whether the inhabitants are affected with goitre or not. In pursuing the enquiry further, it is found that every village in the same neighbourhood is not equally affected, but that some are quite exempt, and others affected to the extent of half their population; and this difference is not found to depend on any accidental or transitory cause, such as usually influence epidemic complaints; but has always affected the inhabitants of a particular village, while those of adjoining hamlets have continued perfectly and permanently free from the complaint." (pp. 265 to 270.)

The primitive district above referred to, constituting the second division of the province, is composed of granite, hornblende slate, gneiss, mica, and clayslate, each of which impresses a particular character on the ranges they compose. The sides are usually precipitous, and covered by a thin and unproductive soil, giving support to a poor and scanty population. The following Table exhibits the result of the detailed observations contained in the second section.

| Districts, and groups of Villages. | Number of high-caste inhabitants. | Number of low-caste inhabitants. | Total. | Number of high-caste inhabitants with goitre. | Low-caste inhabitants with goitre. | Rocks composing the sites of Villages, and in which the Springs are situated. |
|---|---|---|---|---|---|---|
| A. Patan | 200 | 0 | 200 | 0 | 0 | Clayslate. |
| B. and C. Gomedoce | 850 | 100 | 950 | 3 | 1 | Transition-slate. |
| D. Pausall | 700 | 100 | 800 | 3 | 0 | Clayslate. |
| E. Agee | 550 | 50 | 600 | 4 | 0 | Clayslate. |
| F. Bentally | 460 | 40 | 500 | 4 | 2 | Clayslate. |
| G. { Jeercoonee, north side | 200 | 0 | 200 | 0 | 0 | Siliceous sandstone. |
| G. { Ditto, south side | 150 | 50 | 200 | 0 | 0 | Clayslate. |
| H. { Popoulee | 50 | 4 | 54 | 0 | 0 | Hornblende-slate. |
| H. { Rigong | 170 | 26 | 196 | 0 | 0 | Transition-slate. |
| | 3330 | 370 | 3700 | 14 | 3 | |

To the abstract here given might be added a population of about 4,000, who inhabit the gneiss and granite district, and who reside a few months of the year in the plains. These persons are affected with goitre in the proportion of 1 in 500, which would make the whole population of the primitive mountains, embraced by the map, on the south of the Ramesa river, 7,700 souls; of these, about 25 are affected with goitre, or about 1 in 308. Those villages are excluded from the table which have not been inhabited more than nineteen years, or since the period when the province fell into the hands of the British, as also the villages connected with the military posts.

The following is a specimen of the facts of which this table contains an abstract. *b*. Nine villages, situated at a mean height of 4,300 feet, on the N.W. acclivity of a mountain of primitive and transition slate, and having a mean annual temperature of about 64°, contain 800 inhabitants, of whom 4 have small goitres, acquired, they said, in their youth, when residing in a distant part of the country. *c*. Four small villages, on the east of the same mountain, in the hot valley of the Maha-kalee river, at a height of 3,800 feet, has no case of goitre. *g*. On the rugged mountain of Jeercoonee, inclosed by deep ravines, are seven villages, containing 400 inhabitants, without a single case of goitre. The altitude of these villages is from 2,200 to 3,500 feet; but, being exposed to the sun and sheltered from the prevailing winds, their temperature is high. Nothing can be more frightful than the situation of these villages, overhung by lofty cliffs and mountains, while deep chasms lie below.

That this remarkable exemption does not depend on hereditary predisposition, Mr. M'Clelland properly infers, from numerous cases having occurred to him of persons acquiring the disease on removing to an affected district, and from the disease becoming stationary or disappearing entirely during a residence in a healthy village. This is further confirmed by a comparison of the health of 400 troops stationed for three years at Lohoogât, in the part of the province included in the table, of whom not one acquired the disease; while at Petovagur a similar detachment, which entered the hills at the same time, and was in every respect similarly circumstanced, had no goitre during the first year; 5 during the second; and before the end of the third, no less than 16. The former station is in a small valley, 5,562 feet above the level of the sea, having an east and west direction, and surrounded by hills that rise from 300 to 1000 feet above it. The mean temperature is about 60°. Clay-slate and granite rock, covered by a stratum of red ferruginous clay, are the only rocks to be seen; and from the former numerous springs, whose temperature varies at different seasons, issue and supply the wants of the troops. By some experiments recorded in a different part of the work (p. 355 and 259), it appears that the solid contents of the water amounts only to about $\frac{1}{13000}$, and that it is nearly deficient in carbonic and sulphuric acid, iron, and lime, but contains a little alkaline muriate. The waters of eleven springs in the clayslate in different parts of the province have the same properties. Petovagur, fifteen miles north of Lohoogât, stands on the summit of a low ridge, that extends into the centre of Shore valley, throughout which goitre is very prevalent. The general level of the valley is 5,000 feet above the sea, and that of the cantonment 460 feet higher, and separated by several miles of rich cultivated plain from any loftier hills; its situation is, consequently, more open than that of Lohoogât. The mean annual temperature is 1° 40′ higher. The approach to Petovagur passes along a small river valley, over rocks of primary limestone, forming the most frightful precipices that can be imagined. These precipices compose broken and seemingly tottering mountain acclivities, that ascend in some places 3,000 or 4,000 feet; and, as the only road lies along the verge of these cliffs for several miles, it is impossible for the most indifferent traveller to pass insensible either to the danger of his situation or the beauties of the scene, increased by the contrast of the rich valley into which it leads, and from which the ridge of Petovagur rises. This ridge is composed of clayslate supporting extensive deposits of limestone which give a rugged aspect to the surrounding country, the scenery of which is thus described.

"Primitive clayslate forms the basis of this part of the district, and ascends to elevations which are occasionally above 8,000 feet, on which limestones rest, and bestow their particular stamp on the aspect of the neighbourhood. The mountains are here more majestic than in either of the other sections; each individual standing almost detached from the group to which it belongs, and bearing some well-marked character, which leaves on the mind an impression not easily effaced. Thus we find, in the Shore district, every mountain distinguished by some traditional name, derived from a sacred work or ancient temple, which usually caps the summit. At certain festivals crowds of the superstitious population resort to these romantic caves and temples; and on more private occasions the solitary devotee often ends his life, in the attempt to gain an almost inaccessible summit, in order to invoke some grotesque representation of the deity to which the mountain is dedicated. How forcibly the relation of such localities, for religious purposes, attests the influence of what is awful in nature over the mind of man, even in his rude and nearly savage state! In the infancy of civilization (says Humboldt) high places were chosen by the people to offer sacrifices to their gods; the first altars—the first temples were erected on mountains. This remark was suggested by his intercourse with the aboriginal inhabitants of the Andes; its accuracy is confirmed by the customs that prevail among the Hindoos of the Himalayas." (p. 36.)

Copper and iron pyrites are interspersed between the limestone and slate, and disseminated through the strata, seams and rifts of the limestone; while the lower levels of the valley are composed of beds of gravel, the debris of surrounding mountains, cemented by calcareous matter.

The waters of four springs at this station, and of a number of others where goitre prevails, were examined, and nearly agreed in their character. They were copious, less numerous than those in the clayslate, and of a temperature corresponding to the mean of that of the place. The water often rushes from the earth with ebullition, crystalline and sparkling, and deposits immense quantities of calc-tuff; and the rocks around have usually a ruined and broken character. Carbonic acid and lime, with a little iron, and earthy alkaline muriates were detected. No sulphuric acid was indicated by the usual tests.

Mr. M'Clelland has given a statement, showing the admissions from all diseases for three years, into the hospitals of Lohoogât and Petovagur, from which it appears that the sick were nearly twice as numerous at the latter as at the former, and that fever and bowel complaints were one third more prevalent; which circumstance indicates a greater intensity of endemic causes in the district where goitre prevails: an important fact, regarding which we could have wished more information in the statistical details that follow, but which, we are aware, it may have been impossible to obtain.

Throughout the district of Shore and the neighbouring villages, the prevalence of goitre follows, in a remarkable manner, the situation and nature of the strata; the affected villages running along the limestone rocks parallel to the central ridges, composed of clayslate, and which are nearly or altogether exempt from the disease, except in some instances, where the inhabitants are supplied with water from springs, rising in limestone rocks. Mr. M'Clelland enters into many minute details to show that this relation, which is so striking when the grand divisions of the country are considered, is equally so when neighbouring villages are examined; *and even when different parts of the same village, or different classes of the inhabitants use the water of springs issuing from different rocks.* The following Table contains an abstract of these enquiries regarding forty villages (p. 298):

| Paragraph under which the villages are described. | Brahmins and Rajpoots. | Domes (low caste.) | Total inhabitants. | Brahmins and Rajpoots affected with goitre. | Domes affected with goitre. | Total in each village affected with goitre. | Rocks from which the water is derived for the use of the inhabitants of each village. | Rocks on which the villages are erected when different. |
|---|---|---|---|---|---|---|---|---|
| 1 | 60 | ... | 60 | ... | ... | ... | Clayslate | |
| 2 | 25 | ... | 25 | ... | ... | ... | ... ... | |
| 3 | 7 | 7 | 14 | 2 | 4 | 6 | Transition limestone | |
| 4 | 25 | ... | 25 | ... | ... | ... | Clayslate | |
| 5 | 40 | 30 | 70 | 2 | 3 | 5 | | |
| 6 | 10 | 8 | 18 | 7 | 5 | 12 | Transition limestone | Conglomerate of calc.-tuff, slate, and limestone. |
| 7 | ... | 20 | 20 | ... | 7 | 7 | ... ... | Clayslate, coated with calc.-tuff. |
| 8 | ... | 40 | 40 | ... | 2 | 2 | Clayslate | |
| 9 | 48 | 6 | 54 | 3 | 1 | 4 | Limestone and slate | Clayslate. |
| 10 | 20 | 50 | 70 | 3 | 13 | 16 | Limestone (?) | Clayslate, incrusted with calc.-tuff. |
| 11 | 50 | 30 | 80 | 8 | 10 | 18 | Limestone (?) | Clayslate incrusted, with calc.-tuff. |
| 12 | 70 | 20 | 90 | 1 | 6 | 7 | Limestone and slate | |
| 13 | 60 | ... | 60 | ... | ... | ... | Clayslate | |
| 14 | 25 | ... | 25 | ... | ... | . . | ... ... | |
| 15 | 18 | 22 | 40 | ... | ... | ... | ... ... | |
| 16 | 70 | ... | 70 | 2 | ... | 2 | ... ... | |
| 17 | 30 | ... | 30 | ... | ... | .. | ... ... | |
| 18 | 40 | 10 | 50 | ... | ... | ... | ... ... | |
| 19 | ... | 40 | 40 | ... | ... | ... | ... ... | |
| 20 | 100 | ... | 100 | ... | ... | ... | ... ... | |
| 21 | 15 | ... | 15 | ... | ... | ... | ... ... | |
| 22 | 25 | ... | 25 | ... | ... | ... | ... ... | |
| 23 | 100 | ... | 100 | ... | ... | ... | ... ... | |
| 25 | 15 | ... | 15 | 5 | ... | 5 | Transition limestone | Clayslate, coated with calc.-tuff. |
| 26 | ... | 14 | 14 | ... | 7 | 7 | Limestone | Clayslate, coated with calc.-tuff. |
| 27 | ... | 16 | 16 | ... | 10 | 10 | Transition limestone | Conglomerate of calc.-tuff, clayslate, and limestone. |
| 28 | 60 | 40 | 100 | 27 | 33 | 60 | Limestone (?) | ... ... ... |
| 29 | 80 | ... | 80 | ... | ... | ... | Clayslate | ... ... ... |
| 30 | 25 | ... | 25 | 7 | ... | 7 | Limestone | Conglomerate of calc.-tuff, and fragments of slate. |
| 31 | 10 | ... | 10 | 5 | ... | 5 | ... ... | Clayslate (?) |
| 32 | 10 | 15 | 25 | 4 | 9 | 13 | ... ... | Clayslate and calc.-tuff. |
| 33 | 25 | ... | 25 | 4 | ... | 4 | ... ... (?) | ... ... ... |
| 34 | 25 | ... | 25 | 10 | ... | 10 | ... ... (?) | ... ... ... |
| 35 | 24 | ... | 24 | 10 | ... | 10 | ... .. (?) | Conglomerate of calc.-tuff, serpentine, &c. |
| 36 | 40 | ... | 40 | ... | . . | ... | Clayslate | Clayslate and scattered blocks of limestone. |
| 37 | 40 | .. | 40 | .. | ... | ... | Clayslate | Clayslate and scattered blocks of limestone. |
| 38 | 40 | ... | 40 | ... | ... | ... | ... ... | |
| 39 | 100 | ... | 100 | ... | ... | ... | Partial sandstone | |
| 40 | 40 | .. | 40 | ... | ... | ... | Clayslate | Clayslate and magnesian limestone. |
| | 1372 | 368 | 1740 | 100 | 110 | 210 | | |

But this Table will by no means convey a sufficient idea of the minute information on which it is constructed; to some examples of which we must, therefore, request the attention of our readers. 1st. *Beesty*, a village situated on a low group of clayslate hills, in the centre of the valley, has 60 inhabitants (Rajpoots), of whom only one old man has goitre, and he is a stranger, in whom the tumour has diminished since he came to reside in the village. 3. *Salmora*, situated like the former, a little above the valley, but on limestone. Its inhabitants consist of only two families, of different castes, of whom six have goitre, and one (a Rajpoot) is deaf and dumb, with a large head and idiotic expression of countenance. 6. *Goseragong* contains 18 inhabitants, of whom 12 have enormous goitres, and one had died of the disease a few days before Mr. M'Clelland's visit. They assured him that they were generally cut off by the disease before the age of 50. It stands 200 feet above the level of the valley, on a coarse conglomerate of calc.-tuff, and lofty masses of limestone rise abruptly behind it, to the height of 2,000 feet, causing the atmosphere to be confined and hot; but in none of these respects is it more objectionable than the healthy village of Jeercoonee, already mentioned. 9. *Panera.* Of 30 Dome inhabitants using a spring which descends from the limestone cap of a neighbouring mountain, 4 have goitre; while 24 Rajpoots, who obtain it from clayslate, are free from the disease. 12. *Panorah* is built on clayslate, slightly coated with lime, and contains 70 high-caste inhabitants and 20 Domes; of the former, one only is affected with goitre, while six of the latter labour under it. "The Brahmin inhabitants of this village derive their water from a spring in clayslate; and, as the prejudice of the Hindoos denies to the Domes the privilege of partaking of the water of the same spring, the excluded caste is forced in this, as in many other cases in Kemaon, to use this fluid from what they, as well as the Brahmins, believe to be impure sources; and in this instance it is taken from a stream that issues from the same limestone cap that affords water to the two last-described villages." (p. 284.) 13. *Paruree.* This village is situated very low in the valley, on clayslate, affording very fine springs, and no case of goitre exists amongst its 60 inhabitants. The localities of Nos. 17, 18, and 19, amongst whose 120 inhabitants there is no goitre, bear a striking resemblance in external aspect and geological structure to Nos. 25, 26, and 27, of whose 45 inhabitants no less than 22 have goitres. These last are erected on a conglomerate of calc.-tuff, inclosing fragments of clay-slate, and partly on a clayslate hill, which rises 300 feet above them, and is covered by a massive cap of limestone, pouring out numerous fountains, charged with carbonic acid and lime, the water of which is taken for use as it jets from the rock. The altitude, aspect, temperature, religion and morals of both groups of villages are the same, but the former derive their water from springs in clayslate.

The account of No. 28 we shall give in Mr. M'Clelland's own words:

"*Deota*, a lengthened village, which occupies half a mile of the foot of Durge mountain. One extremity of it is inhabited by Brahmins, the other by Rajpoots and Domes. Of the first caste, there are about 20 persons, all of whom are free from goitre; there are 40 of the second, and two thirds are affected more or less; and of the third caste, 46 in number, nearly the whole are affected. To what cause can we ascribe the immunity of one caste of the inhabitants of this village, and the almost universal affection of the other two castes? They are all alike well fed, and have little toil: their land producing the requisites of life almost without labour. Difference of caste does not here imply a difference of pecuniary circumstances, and, consequently, of the comforts of life. In these respects the three castes in this village are on perfect equality; nor will hereditary predisposition, acquired by intermarriages, be sufficient to explain the interesting fact; for the affected parties are confined to the Rajpoots and Domes, who cannot intermarry, while the Brahmins and Rajpoots may. The village is raised about 100 feet above the level of the valley, and the mountain, at the foot of which it is situated, rises with a gentle slope, and is not, in this vicinity, at all rugged. It is chiefly composed of transition limestone, and the village is erected on a conglomerated rock, composed of calc.-tuff, inclosing fragments of other rocks. There is a spring situated in the valley, about 100 yards from the village, which, from its first appearance, has the character of a mineral spring. The water bursts forth with strong ebullition, in the quantity of at least 40 gallons a minute, and agglutinates the sand and gravel by which it is surrounded by the deposition of calcareous tuff. The temperature and quantity of the water is the same at all seasons. The former inhabitants of this village, aware, perhaps, of the noxious effects of the spring, had an aqueduct formed, by which water is conveyed into the Brahmin portion of the village, from a distant source. The aqueduct being allowed to go out of repair, the quantity of water it transmits is reserved exclusively for the Brahmins; but during the rainy season, when water is plentiful, the Rajpoots also use the water of the aqueduct; but the Domes have no alternative at any season but to use the water from the spring." (pp. 285-292.)

Mr. M'Clelland has omitted to state from what rock the water of the aqueduct is derived; but we infer from what follows that it is from clay-slate. 30. The accidental circumstance of a stream depositing calc.-tuff being conducted to the village by a circuitous route to irrigate the fields, seems to have lessened the prevalence of the disease. Nor are the Brahmins exempt when they derive their supplies of water from the limestone; No. 31, containing 10 inhabitants of this caste, having one half affected with the disease. 32. *Oliel.* Two small hamlets, situated in a most pleasing amphitheatre of limestone mountains. Of 25 inhabitants, 13 have goitre; of whom 10 *are cretins, and of these a whole family are deaf and dumb; they seemed also deficient in sight, and quite unsusceptible of the passions of joy and fear.* 35. *Gooraght,* partly built on clay-slate and partly on a calcareous conglomerate, containing with other rocks blocks of *serpentine.* Of 25 inhabitants, 10 have goitre, *and a father and two sons are cretins: the two sons are deaf and dumb.* 40. *Deoneolla.* Situated in the lower part of the valley, partly on clay-slate, and partly on *magnesian limestone, and surrounded by high mountains: had no case of goitre.*

We regret that Mr. M'Clelland's work does not furnish the means of instituting a comparison of the prevalence of bronchocele in villages situated on common limestone, and on the dolomitic rocks he has described (but of which he has given no analysis), as we might thus have had more light thrown on a suggestion of Dr. Inglis's, that the latter is the rock chiefly concerned in producing the disease.

"I cannot say," observes Dr. Inglis, "that in all lime districts, goitre prevails; on the contrary, I know that it does not in many places where the *blue vitrified mountain limestone* is very abundant. Nevertheless, I think that I am correct in supposing that the presence of the magnesian limestone always predicts the co-existence of the disease. Take, for example, that ridge of magnesian limestone running from north to south, through the centre of Yorkshire and margining the shires of Derby and Nottingham. All along that line we have goitre to a very great extent, whereas, on our diverging to either side, the disease is found to diminish. The towns situated on this ridge are Nottingham, Chesterfield, Rotherham, Ackworth, Pontefract, Abberford, Wetherby, Knaresborough, Boroughbridge, and Ripon. After this the magnesian limestone dips, then reappears in the county of Durham; it continues its course almost due north from Darlington to South Shields, where at Tynemouth it meets the sea. In many of these towns I know goitre prevails; *in the others, I should suppose it did,* excepting *where we approach within the influence of the sea, when the morbid action would be counteracted,* for we found before, from Dr. Richardson's statement, that the disease itself was removed by a sea voyage." (p. 23.)

The vagueness of the expressions quoted in italics, and several of the places mentioned not being situated on the magnesian limestone, do not justify us in placing much reliance on these opinions, especially as Dr. Inglis has instituted no comparison between these places and the chalk valleys of Hants and Surrey, and of those parts of Devonshire, and other counties, where there is none of this rock, and where the disease is nevertheless very prevalent, nor are such general assertions as the following properly admitted into enquiries like the present: "Amongst the Alps, in greatest perfection, is met with that double carbonate of magnesia and lime, called Dolomite," of which the snow "water must doubtless carry with it a portion." (p. 15.) Yet the prevalence of the disease, along the upper division of the Lake of Como, where Dolomite rocks are extensively distributed, renders it important to obtain accurate statistical details regarding these very accessible localities. At Colico, for instance, which stands on an unhealthy swamp near the mouth of the Adda, almost the whole inhabitants are afflicted with goitre, while those of other towns are comparatively healthy.

The result of Mr. M'Clelland's enquiries, in various other valleys, is contained in a table, (p. 310;) but we must content ourselves with noticing one or two of the most important observations contained in this section. The valley of Kalapany is amongst the lowest inhabited places in Kemaon, closely surrounded by lofty mountains, and the presence or absence of goitre is marked by the same circumstances as in Shore valley: thus, in the village of Beechelly, having 70 inhabitants, there are 30 persons affected with bronchocele; this village is closely surrounded by mountains of transition limestone, and is erected on alluvial soil, cemented by calc-tuff; while Remina, a mile distant, on slate, has only one case out of 50 inhabitants. In the wild valley of Roilputty there are only two villages, containing 25 inhabitants each, and both situated on slate-rock. The first derives its water from a stream falling over limestone precipices, and a third of the inhabitants approach to the condition of cretins, six of them having goitre; the second, which derives its water from the same stream, one mile and a half farther down, has only one goitre. In this instance Mr. M'Clelland has neglected to communicate the requisite information, as to the course of the stream previous to its entering on the plain, by a short course, through which it is supposed to have lost its noxious qualities.

We shall conclude with a short notice of the valley of Baribice, elevated 4,000 feet above the sea. The eastern extremity is composed of *clayslate,* and in five villages, *containing* 152 *inhabitants, there is not one goitre.* The other extremity of the valley is *partly composed of limestone, and of* 192 *inhabitants, distributed in six villages,* 70 *are affected with goitre*; but Ducygong, one of these villages, *supplied with water*

*from clayslate, has not a single case of the disease; while Ager, only half a mile distant, and containing* 50 *inhabitants, has no less than* 40, *and of that number* 20 *are cretins.* They use the water which issues from an old copper mine in limestone, which contains carbonates of lime, soda, &c., but no sulphate: they were earnestly solicited to discontinue its use, and to employ that of a spring at a little distance, in the full confidence of their being benefited by the change. (p. 304.)

These unfortunate beings, oppressed by their owners or superiors, are reduced to labour in the mines, from infancy to old age, for an allowance hardly sufficient to support life; and the drifts of these works are so low, that the ore can only be dragged out by children, who are, for the most part, frightfully deformed, (Chapter on Mines, p. 168;) a state of things, however, which exists elsewhere in the province, without producing the same effects.

Mr. M'Clelland has devoted a chapter to the circumstances in which cretinism occurs in the Kemaon, which is the more necessary, as Dr. Inglis has stated, apparently on the authority of Mr. Bramley, that cretinism is quite unknown throughout the Himalayan regions, although, in many places, 48 out of 50 inhabitants are goitrous, (p. 94;) and on this has grounded an argument for the distinctness of the diseases, and of their causes. He has ascertained that where goitre prevails, *to a certain extent*, the people exhibit a want of enterprise and bodily vigour, as compared with their immediate neighbours, who are exempt from the disease; and the distinction increases, " not always, but in general, with the extent and severity of goitre," until at length the body is found generally deformed, the head often of enormous size, the spine distorted, and the limbs short and crooked; or swellings of the elbows, knees, or other joints, take place; as also of the lymphatic glands, especially those of the lower part of the neck, and perhaps of the thymus; showing the disease to be complicated or connected with a strumous habit. More frequently the deformity is confined to the head; the features are bulky, and more or less vacant and idiotic, and all the sensations are blunted, yet the individuals seem in general to retain some portion of intellect, but their minds seem never to advance beyond the state in which they were in early infancy, during which this form of the disease always commences, generally about the third year. Congenital swellings of the neck (and sometimes of other parts, as the lobe of the ear,) are observed in animals in Kemaon; and Dr. Campbell has described 22 examples of this in Nipaul, in 17 of which the tumours were composed of cells, containing a glairy fluid, and in five, had a glandular structure. Dr. Campbell has known this to occur in the young of healthy goats and sheep, brought from parts of India, where goitre is not known, (*Calcutta Transactions*, vol. vii.) Both authors are of opinion that it is never congenital in the human subject, but Mr. Bramley and Mr. Baillie Fraser, in his instructive work on the Himalayas, assure us that it is so, and that they have seen it in the youngest infants, a fact supported by the statements of Dr. Inglis and other European authorities, and of which we consider Dr. Campbell's interesting paper to afford a striking confirmation.

Such are some of the facts on which Mr. M'Clelland grounds his opinion, that the predisposing cause of goitre is the use of water impregnated with calcareous salts; and he states that in the course of his personal enquiries, extending over 1,000 square miles, without a view to any theory, no instance has occurred, in which goitre prevails to any extent, where the villages were not situated on or close to limestone rocks. But while we admit the force of these facts, and admire the industry with which they are collected, we are not prepared to admit, without more extended enquiry, conclusions so important, and which leaves unexplained the fact, that the disease is nearly unknown in many districts where lime abounds in the waters, as amongst the poor peasantry of the Roman states, where marble is deposited amidst the foam of the cascades of Tivoli and Terni, or where the sluggish Sarnus and Salso, loaded with lime, pass through the rich and salubrious territory of Naples, or the pestiferous environs of ancient Pæstum. Our own island affords admirable opportunities for investigating the subject, and we are therefore glad of the appearance of Dr. Inglis's pamphlet, as showing that attention has been directed to it, and because this pamphlet contains some valuable facts relative to the prevalence of the disease in several of our towns and districts, and at various periods of life. We are constrained, however, to observe, that we shall expect, in the promised continuation of the enquiry, more caution and greater minuteness of detail; and would recommend Dr. I. to prepare himself for the investigation, by acquiring the requisite information on the geology of our island, without which he must constantly fall into error. We have already alluded to the careless manner in which he refers to the towns in which bronchocele either prevails or in which *he supposes it does;* and in the appendix communicating the result of new investigations, the absence of goitre from the northern counties of Scotland is thus noticed: "Lime, to a considerable extent, exists in these counties; but, as in other parts of Scotland, it is the *pure mountain*, and not the magnesian limestone." (p. 79.) We are at a loss to understand how the great siliceous and calciferous conglomerates and sandstones, with their subordinate beds of cornstone, of the old red sandstone formation, so extensively distributed over these counties, and so well described by some of our most eminent geologists, or the primitive limestones of the Grampians, could have been confounded with the mountain or carboniferous limestone. If such gross errors find their way into medical works relative to Britain, the geology of which is of all countries best known and most accessible, what hope have we of advancing one step in any professional enquiry connected with other physical sciences? Equally futile is the analogy drawn between the coal formation of England and the tanks of India, as producing goitre.

In the prosecution of his enquiry, in addition to endeavouring to ascertain the whole of the physical and moral circumstances of the inhabitants of each place; we would suggest to Dr. Inglis a particular investigation of the supposed influence of the sea-air in preventing the disease, of which we have heard stronger proofs than the fact quoted from Dr. Richardson; and whether any impregnations, such as iodine, exist in certain waters, to which exemption may be ascribed. The details of analysis of waters should also be put on record, when these have been performed by chemists of known accuracy. But to do all this with any prospect of advantage, the author must give up all idea of writing "in some measure, as well for the people as the profession," regarding a disease for which he proposes no method of prevention, and recommends no remedy but iodine: in this, however, he has so much confidence, and thinks it so safe, that he would wish to see "the people" employ it without medical advice.*

We shall now turn to Mr. Bramley, whose position, as surgeon to the ambassador at Nipaul, offered many facilities for extending his enquiries into the surrounding regions, through the numerous travellers who meet in this intermediate post between the east and west of Asia. This lamented physician has the glory of having founded, and almost matured, the first medical school in which the natives of Asia are instructed in all branches of European medical science; and his varied acquirements and his knowledge of bronchocele as it occurs in Switzerland, peculiarly qualified him for the enquiry, and has enabled him to produce a most instructive memoir. The style, however, is too diffuse, and the observations of authors are occasionally so mixed with his own, as not easily to be distinguished from them; nor has he attempted to account for the striking facts he records of villages where goitre is very prevalent being situated close to others in which it is unknown, and which irresistibly forces us to refer the disease to a local cause. The distribution of rocks in Nipaul are more simple than in the neighbouring district of Kemaon, and we hope that the present distinguished resident,† or some of the gentlemen attached to his mission, will include this enquiry in their other important researches into the natural history of this most interesting district. Mr. Bramley enlisted the chemical skill of Mr. Prinsep, in an enquiry into the composition of some of the waters of Nipaul. Sulphate of lime is almost entirely absent from the whole, and the carbonates are by no means abundant; but there is no information as to the precautions taken to prevent the escape of the carbonic acid, and the deposition of lime, previous to the analysis, nor of the prevalence of goitre amongst the inhabitants using them. Of the seven waters examined, however, one, which contains only a little muriate of soda and a trace of iron, is from Sanchoo, a considerable town, situated on an eminence in the valley of Nipaul, and surrounded by a luxuriant forest, in which it appears, from another part of the paper, that goitre does not occur although very prevalent in the surrounding villages: the Nipaulese also universally ascribe the disease to the water. (Compare p. 188 with table, p. 202.) Mr. Bramley did not find goitre much more common amongst women than men, but in the latter the tumour was often small, and frequently unnoticed by the subjects of it: of 65 goitres in the town of Handygong, 29 were in men, 31 in women, and 5 in children; and in Deopatan, 34 were in men, 38 in women, and 11 in children. He confirms the general opinion that the periods of adolescence and of parturition favour the development of the disease; thus, in 124 males and 140 females, 15 of each were under ten years, a proportion not very different from that given by Dr. Inglis, from an excellent report by Dr. Paley, of Bishoptown.

* Surely Dr. Inglis does not mean the assertion that goitre is as prevalent in some parts of Yorkshire as in any of the Alpine villages to be taken literally; and why allege, as a reason for addressing his book to the people, that the disease is "evidently on the increase" (Preface), of which no proof is adduced? A good account of the disease, as it prevails to the north of the Dartmoor hills, while the southern limestone tracts are exempt from it, would be a valuable addition to our knowledge.

† Colonel Hodgson, the distinguished zoologist and orientalist.

Bronchocele has been ascertained to prevail from the left bank of the Ganges, in the plains of Hindoostan, to the northern boundaries of Tibet, from Assam,* in the 27th degree of north latitude, and 93d of east long., the northern boundary of Oude to Huredwar, in 30° north latitude, and 78°, 25′ east long. It occurs, along with cretinism, beyond the great wall to the N.E., and at Yarkmid to the N.W. of China, and, as Mr. Bramley was informed by Tibetan travellers, in the Mongolian and Tibetan countries lying between them; and it may be traced in various places near the lake of Baikal, and on the Lena. Over such vast countries all varieties of climate prevail, and the food of the inhabitants differs from a nearly pure farinaceous diet to one almost exclusively animal. To show this more distinctly, Mr. Bramley divides the country, to which his personal observations or enquiries extended, into three districts, of which the first, extending from the Ganges to the mountains, consists of two parts, the one richly cultivated and salubrious, partaking of the seasons common to the rest of India, and whose inhabitants may be regarded as a healthy race of men, having the common physical attributes, and living on the ordinary food of Hindoos and Mussulmans. Goitre occurs at Tirhoot and other districts of this tract; and Mr. Evans mentions that European children recover from it on being removed to situations where it is not prevalent. The other portion of this tract is that dismal region called "Terai" (the moist), which bounds the plains, and in which an excess of vegetation and humidity, and the want of ventilation, caused by the dense forests that cover it, prove most destructive to health and life; and while it prevents the access of strangers, causes the inhabitants to have a mean and stricken appearance, and to be debased in form, size, and strength. Amongst them, Mr. Bramley states the disease to be very common, but in this he differs from Mr. M'Clelland, whose observations, however, refer to a different part of this unhappy tract. The second region is that central mountainous country, including Nipaul, Kemaon, &c., between the plains of India and the elevated table-land of Tibet, consisting of irregular masses of very precipitous mountains, varying in elevation from 500 to 25,000 feet, and in some places inhabited at an elevation equal to that of Mount Blanc. It is subject to temperatures as various, both in changeableness and intensity, as its infinitely diversified elevations and physiognomy; for while the sugar-cane flourishes in the lower valleys, the cerealia disappear from the higher elevations. The distribution of the seasons is tropical, but influenced, even in the valleys, by the cold blasts from the higher mountains; its humidity is less than that of Bengal, and greater than in Europe, and at the end of autumn fogs prevail. The soil is deep, produces abundance of good grain, and the people live in tolerable ease, and consume a good deal of animal food, especially the aboriginal and inferior tribes. Goitre prevails very extensively, particularly amongst the poorer classes, and on the borders of the great valley at an elevation of from 500 to 5,000 feet above it; as, at Phirphen 15 per cent., "Chitlong 40, and at a small village, named Chirphu, on the crest of a high mountain, 48 individuals had the disease out of a total of 53. These were all counted by myself, and included men, women, and children, excepting only those in the

* Leslie on the medical topography of Gomattee, in Lower Assam. (Calcutta Medical Transactions, vol. vi.)

arms." (*Bramley*, p. 186.) And natives, coming from those parts of India where the disease does not prevail, become subject to it, to the extent, even at the capital, where it is comparatively rare, of 15 per cent. per annum.

The Trans-Himalayan region, to which these remarks apply, is a vast and nearly uniform plain; the soil is sandy, and like that of Bischir, where Mr. B. Fraser found the disease so common, is much impregnated with salt. It is a bare, bleak, cold, and dry region, subject to high winds, but salubrious; and the inhabitants, who are remarkably athletic and muscular, live almost entirely on animal food, which is very abundant. The prevalence of goitre amongst these people is a remarkable fact, when contrasted with the authentic statements, that at a certain height above the Vallais, cretinism and goitre disappear; and the important observation of Marsden, that it is only known in Sumatra amongst the deep valleys subject to dense morning mists, and is cured by removal to the clear and pure air of the higher summits. Can this difference be accounted for by the peculiar geological structure of the Himalayas, in which limestone, in some places abounding in fossils, reaches above the highest inhabited spots; while in the Alps, especially in the Vallais and along the Arve, primary rocks form the sides of the higher valleys. ?* At Geneva, certain wells, now filled up, excavated in a recent *calcareous* sandstone, the waters of which abounded in sulphate of lime, were known to produce the disease in a few weeks or months in natives and in the French soldiers formerly quartered near them, who were exempt from it while they drank the nearly pure water of the lake. At Cluses, on the Arve, also 1,600 feet above the level of the sea, numerous cretins and goitrous persons are seen in the streets; lofty cliffs of limestone tower over the town, and through its caverns copious streams of water find a passage; higher in the valley, goitre becomes rare, and the granite of Mount Blanc, gneiss, and mica slate form the prevailing rocks. In ascending from the Vallais along the Val Valorsine we find similar rocks with clay-slate and the peculiar siliceous conglomerate called "poudingues de Valorsine," to succeed the calcareous strata of the Rhone. So it is in Westmoreland and the Grampians, where goitre is unknown. In the south of India, also, the Neilgherry mountains, rising 9,000 feet from the plains, and enjoying a climate like that of Europe, are exempt from the disease; as are the narrow and deep valleys of Coong, closed in on every side by precipitous mountains, from 3,000 to 5,000 feet high, covered with dense forest, or rising in magnificent precipices. In 1835-6, at Mercera, the capital of Coong, 119·14 inches of rain fell; the mean temperature was about 65°, the quantity of moisture 188, and the dew point 11·4. These countries are entirely composed of syenite and other primary rocks, and no lime is found in the Neilgherries.† It should however be observed, that bronchocele is extremely rare in the south of India even where lime-

* In the journal of a tour in the Himalayas published in the Asiatic Journal for August (p. 240), the inhabitants of the district of Gurhwal are stated not to suffer from goitre. The rocks seen were mica slate, quartz, and quartz-ore sandstone.

† The medical topography, meteorology, and geology of the Neilgherries, and also much information regarding Coong has been published by Dr. Baikie in a separate work, also in various papers by him, Dr. Benra, &c. in the Calcutta Transactions and in the Madras Quarterly Journal of Science. Meteorological science is cultivated with great success in India; yet, notwithstanding the opinions of Sir John Herschel and the British Association, such writers as Dr. Prout, &c., continue to quote foreign writers for uncertain accounts of the climate of these regions.—See Bridgewater Treatise on Chemistry and Meteorology.

stone abounds; but this rock rarely attains a great elevation, is often impregnated with salt, and does not often afford water to the inhabitants. The case of a lady, however, has been mentioned to us, who twice contracted the disease in the hilly country of Travancore where fossiliferous rocks, probably tertiary, occur; and recovered on returning to Tranquebar on the eastern coast. The people of Tibet are said to suffer little from scrofula, and rachitis is nearly unknown in Nipaul, yet Mr. Bramley is of opinion, that the predisposing cause of the disease is often a want of tone in the living fibre, and some defect in the lymphatic system connected with a strumous habit; an opinion not very different from that still more strongly advocated by Mr. M'Clelland.

Of the treatment of bronchocele we have space to say little. Mr. M'Clelland places in a proper light the distressing and even fatal consequences of the disease, and its injurious effects on the head, heart, and organs of voice and respiration; regarding which, strange misconceptions have found their way into various works. No mention is made of extirpation of the gland before it has attained a great size, as noticed by Mr. Fraser; an operation, the performance of which by Hindoos we might have been inclined to doubt, notwithstanding the recent accounts of their practice of lithotomy and extraction of the crystalline lens, had not Mr. Fraser kindly informed us, that he could not have been deceived in the information he received, and that the scars he saw could not have arisen from the actual cautery, even if that could be supposed capable of removing the disease. The use of protection and support to the throat is strongly recommended by Mr. Bramley, who, besides the notice of several cases successfully treated by this alone, adduces the singular fact of the Hill Coolies, who carry loads over the mountains in baskets attached to straps passing over the forehead, which causes an unusual development of the cutaneous muscles of the throat, never having the disease of any size. It deserves to be noticed, however, that this does not prevent altogether the enlargement of the gland, which spreads under the thickened muscular fibres. Stimulating frictions alone will occasionally succeed, and Dr. Campbell, Mr. Bramley's successor at Nipaul, found Croton oil very useful; but both pressure and friction have failed where iodine ointment has effected a rapid cure. The most common variety has the form of a shapeless protuberance consisting of a congeries of cells, occurring chiefly in women of feeble habit, and attaining a large size, although its growth is slow. Its cure is tedious and difficult; indeed Dr. Campbell asserts that he has seldom been able to remove it even by iodine, (*India Journal of Medicine*, Jan. 1837,) and we are sorry to observe that a similar statement is made by Dupuytren in his clinical lectures. Mr. Bramley was either more fortunate or more persevering, as he seldom failed to relieve the patient; and in one case not only did the enlargement of the gland disappear, but the neck became shriveled from the absorption of the subcutaneous adipose tissue. When the tumour is pulsatory, the whole of the vessels of the part partake of the increased action, and are remarkably increased in size; it has often an inflammatory character, and may arise from local injury, or the suppression of habitual discharges, but seldom occurs before the age of puberty. Depletory measures will often check this affection, and it yields very quickly to the internal use of iodine. In one case in which this remedy was given in the form of tincture and produced alarming constitutional disturbance, the tumour was discussed in six days, during two of which the medicine was omitted. When we see a disease like this, in which the vessels are not only excited to undue action, but absolutely enlarged, removed in so short a time by a remedy of such recent discovery, we cannot but indulge the best hopes of the progress of medicine. Dr. Prevost, of Geneva, extirpated one of the thyroid glands of a dog affected with goitre (in this animal the two lobes of the gland are distinct), and found the vessels greatly enlarged; and then administered iodine for the cure of the other; in a short time the swelling was removed, and on dissection, the enlarged vessels were found almost obliterated. The lymphatic variety is smooth, firm, and elastic, seldom attains a large size, and occurs most frequently in men. It is easily cured by the local application of iodine. Of 116 cases of all kinds treated by Mr. Bramley, only three or four occurred in which the patients were not either cured or so much relieved as to show that perseverance in the use of the remedy would have been successful: and these were old persons in whom the disease was of long standing, and in one of cartilaginous hardness. Mr. Bramley makes some good observations on the effects of the internal use of iodine on some constitutions, viz., vascular excitement, palpitation, anorexia, tremors, diarrhœa, difficult respiration, absorption of glandular structure, &c., proceeding to an alarming extent, and not easily counteracted, or their occurrence accounted for, as other patients take larger doses without their having any effect. We believe that aggravated examples of the same kind are often produced by the indiscriminate use of the preparations of iodine in this country for all obscure or obstinate affections; and we have witnessed its abuse so often, that we are little surprised that some of the most eminent physicians and surgeons now discourage its use, even in cases for which it certainly forms a most valuable remedy.

We have greatly trespassed on the limits that some of our readers may think should be assigned to such a subject, but we could not allow ourselves to doubt, that the minute and careful researches of our oriental brethren into a disease prevalent more or less in every region of the earth, evidently connected with local causes, and presenting peculiar facilities for the study of the action of external agents on the bodies of men and of animals exposed to them, would be interesting to many both in England and on the continent. It is not often that individuals so variously gifted for pursuing the enquiry, will visit those magnificent but often inaccessible regions; and Mr. M'Clelland has unfortunately chosen a form of publication which prevents his work ever reaching the medical libraries of this country. It is also true, that of the Transactions of the Calcutta Medical and Physical Society, only a few copies are to be found in England, and we believe not one in the whole of those parts of the continent where bronchocele is endemic. We lament that the Medical Society of Calcutta has substituted a medical journal of a miscellaneous character, placed entirely under the control of the secretaries, for the Transactions previously published under the authority of a "committee of papers;" as this plan must place still more out of the reach of their European brethren the labours of our countrymen in the east, by mixing selections, reviews, and matters of merely temporary interest with original communications.

